# Humanized Mice for the Evaluation of Novel HIV-1 Therapies

**DOI:** 10.3389/fimmu.2021.636775

**Published:** 2021-04-01

**Authors:** Shawn Abeynaike, Silke Paust

**Affiliations:** ^1^ Department of Immunology and Microbiology, The Scripps Research Institute, La Jolla, CA, United States; ^2^ The Skaggs Graduate Program in Chemical and Biological Sciences, The Scripps Research Institute, La Jolla, CA, United States

**Keywords:** humanized mice, BLT, DRAG, HIV-1 infection, viral latency, latency reversal, immunotherapy, gene therapy

## Abstract

With the discovery of antiretroviral therapy, HIV-1 infection has transitioned into a manageable but chronic illness, which requires lifelong treatment. Nevertheless, complete eradication of the virus has still eluded us. This is partly due to the virus’s ability to remain in a dormant state in tissue reservoirs, ‘hidden’ from the host’s immune system. Also, the high mutation rate of HIV-1 results in escape mutations in response to many therapeutics. Regardless, the development of novel cures for HIV-1 continues to move forward with a range of approaches from immunotherapy to gene editing. However, to evaluate *in vivo* pathogenesis and the efficacy and safety of therapeutic approaches, a suitable animal model is necessary. To this end, the humanized mouse was developed by McCune in 1988 and has continued to be improved on over the past 30 years. Here, we review the variety of humanized mouse models that have been utilized through the years and describe their specific contribution in translating HIV-1 cure strategies to the clinic.

## Introduction

Human immunodeficiency virus-1 (HIV-1) was first discovered in 1983 by the laboratory of Luc Montagniers at the Pasteur Institute, by culturing T cells isolated from the lymph nodes of a patient with early symptoms of AIDS (1). Since its discovery, the battle to control and eradicate HIV-1 has been long and tumultuous. Close to four decades have passed. While great strides have been made in managing HIV and preventing the onset of AIDS in patients *via* daily treatment with combination antiretroviral therapy (ART) (2), a successful vaccine or curative treatment has yet to be developed. The key to research on HIV cure therapy is using a suitable animal model, as a comprehensive analysis of the human immune system is limited due to ethical and practical restrictions. Humans and chimpanzees are the natural hosts for HIV-1 replication. However, due to ethical and practical reasons are not amenable to most methods of experimentation. Mice reconstituted with human immune systems and non-human primates are the two animal models that have received the most attention in investigating HIV-1 pathogenesis. Non-human primate models provide many advantages, such as being a natural host for the closely related Simian Immunodeficiency Virus (SIV) and the chimeric SHIV virus and having similar anatomical and physiological features to humans. The complete range of advantages and disadvantages of these models have been reviewed elsewhere (3–5). In contrast, humanized mice contain human CD4+ T cells, which are permissible to HIV-1 infection and simultaneously allow in-depth analysis of the human immune response to HIV-1 pathogenesis *in vivo*. Humanized mice have provided important insights into preventative approaches to HIV-1 infection (6–8). These approaches have been reviewed in detail elsewhere (9, 10). Here we review the history and development of humanized mouse models (**Tables 1** and **2**) and describe their applications in a wide range of novel approaches for HIV-1 eradication (**Table 3**).

**Table 1:** Summary of humanized mouse models and their tissue-based chimerism.

	SCID-hu	hu-PBL	hu-HSC	BLT	TKO-BLT
**Mouse Model**	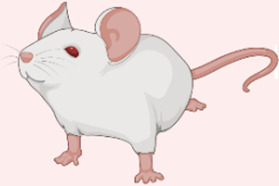	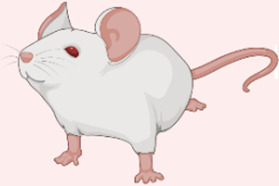	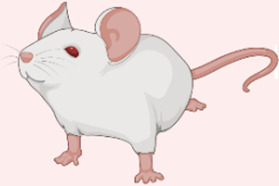	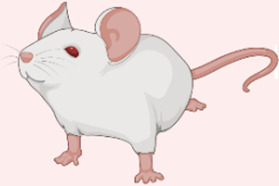	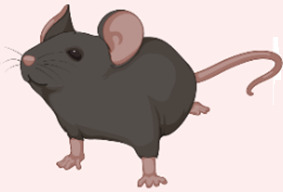
**Genetic Background**	C.B17scid/scid (SCID)	SCID NOD-SCID NSG BRG NCG	SCID NOD-SCID NSG BRG NRG DRAG	SCID NOD-SCID NSG NRG	C57BL/6 Rag2 ^-/-^ $g_{c}^{-/-}$
**Humanization Method**	Subcapsular Coimplantation of human fetal thymus and liver fragments	Intraperitoneal Injection of human PBMCs	Injection of CD34+ cells from cord blood/fetal liver	Coimplantation human fetal thy/liv with i.v. injection of CD34+ cells from fetal liver	Coimplantation human fetal thy/liv with i.v. injection of CD34+ cells from fetal liver
	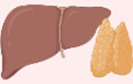	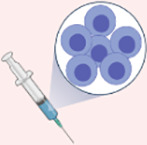	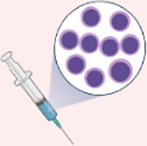	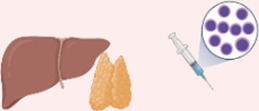	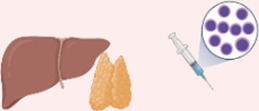
**Immune Reconstitution**	T cell engraftment Multilineage hematopoiesis No primary immune response	T cell engraftment No multilineage hematopoiesis No primary immune response	Multilineage hematopoiesis Primary immune response No HLA restriction	Multilineage hematopoiesis Primary immune response Human HLA T cell restriction	Multilineage hematopoiesis Primary immune response Human HLA T cell restriction
**References**	McCune Namikawa (11), Namikawa Weilbaecher (12)	Moiser, Gulizia (13), Hesselton. Greiner (14), van Rijn, Simonetti (15), Ali, Flutter (16)	Kamei-Reid and Dick (17), Peault, Weissman (18), Hiramatsu, Nishikomori (19), Danner, Chaudhari (20)	Lan, Tonomura (21), Melkus, Estes (22), Brainard, Seung (23), Stoddart, Maidji (24)	Lavender, Messer (25), Lavender, Pang (26), Lavander, Pace (27)

Created with BioRender.com.

**Table 2:** Summary of known immunophenotypic characteristics of humanized mice.

Humanized mouse model	Immune cells	Phenotypic characteristics	References
**Hu-PBL**	T cells	Donor immunological memory is conferred	Mosier, Gulizia (13); Hesselton, Greiner (14); van Rijn, Simonetti (15), Ali, Flutter (16)
		High activation due to MHC mismatch and thus high propensity of GVHD	
	B Cells	Present at low levels	
		Donor immunological memory is conferred	
		Limited class switching and SHM	
**Hu-HSC**	T cells	HLA mismatched T cells	Kamel-Reid and Dick (17); Péault, Weissman (18); Lapidot, Pflumio (28); Hiramatsu, Nishikomori (19); Shultz, Saito (29); Danner, Chaudhari (20); Choi, Chun (30); Chen, He (31); Ito, Takahashi (32); Rongvaux, Willinger (33), Billerbeck, Horwitz (34), Billerbeck, Labitt (35);Ding, Wilkinson (36); Majji, Wijayalath (37); Li, Mention (38); Herndler-Brandstetter, Shan (39)
		Th2 Polarization driven by increased GATA3 expression	
	B cells	Predominantly immature, express CD5+ at higher levels	
		Limited class switching and somatic hypermutation	
		IgG and IgM present at lower levels than in humans	
	NK cells	Present in low numbers	
	Macrophages	Present and intact phagocytic function	
	Dendritic Cells	Both plasmacytoid DCs and Myeloid DCs are present at low frequencies	
		Able to induce the activation of allogeneic human T cells	
		Able to produce IFN-a upon stimulation	
	Mast Cells	Present in low numbers	
		Incomplete development due to lack of species-specific cytokines	
		Poor allergic responses	
**BLT**	T cells	HLA restricted T cells	Lan, Tonomura (21); Melkus, Estes (22); Brainard, Seung (23); Stoddart, Maidji (24); Biswas, Chang (40); Kalscheuer, Danzl (41); Denton, Nochi (42), Smith, Lin (43); Bryce, Falahati (44), Honeycutt, Wahl (45); Nikzad, Angelo (46)
	CD8+ T cells	Capable of functional, antigen specific effector responses	
		Tend towards a more naïve phenotype expressing high levels of CD45RA, CD27 and CCR7	
	CD4+ T cells	Capable of functional, antigen specific responses	
		Th2 Polarization driven by increased GATA3 expression	
	B cells	Limited class switching and somatic hypermutation	
		Incomplete maturation indicated by CD10+ B cells in Spleen and Bone Marrow and CD5+ B Cells in spleen and peripheral circulation	
		IgG and IgM present at lower levels than in humans	
	NK Cells	Lower numbers but phenotypically similar to human NK cells	
		Maintain cytotoxic activity	
		CD62L expression is higher in spleen and liver potentially caused by underdeveloped lymphoid follicles	
	Dendritic Cells	Both plasmacytoid DCs and Myeloid DCs are present at low frequencies	
	Macrophages	Present and intact phagocytic function	
		Incomplete development due to lack of species-specific cytokines	
	Mast Cells	Present in spleen and lung	
		Absent in skin, heart, stomach, and small intestine	
		Incomplete development due to lack of species-specific cytokines	
		Poor allergic responses	

**Table 3:** Summary of therapeutic approaches and humanized mouse models utilized in their testing.

Therapeutic approach	Model	Genetic background	References
bNAbs	Hu-PBL	SCID	Poignard, Sabbe (47)
	Hu-HSC	NRG	Klein, Halper-Stromberg (48), Horwitz, Halper-Stromberg (49), Halper-Stromberg, Lu (50)
		NSG	Wang, Gajjar (51)
	BLT	NSG	Badamchi-Zadeh, Tartaglia (52)
CAR T Cell Therapy	Hu-HSC	SCID	Kitchen, Bennett (53)
	BLT	NSG	Kitchen, Levin (54), Zhen, Kamata (55)
	Hu-PBL	NSG	Leibman, Richardson (56), Bardhi, Wu (57), Anthony-Gonda, Bardhi (58)
Zinc Finger Nucleases	Hu-PBL	NOG	Perez, Wang (59)
		NSG	Yi, Choi (60), Wilen, Wang (61), Yuan, Wang (62)
TALENs	Hu-HSC	NSG	Llewellyn, Seclén (63)
CRISPR/Cas9	Hu-PBL	NCG	Xiao, Chen (64)
	Hu-HSC	NSG	Dash, Kaminski (65)
	BLT	NSG	Yin, Zhang (66)
Block and Lock	BLT	NSG	Kessing, Nixon (67)

## Humanized Mouse Models

### SCID-hu Mouse

Severe combined immunodeficiency (SCID) is a debilitating disease characterized by T and B lymphocyte differentiation impairment. Those affected show high susceptibility to recurring infections from viruses, bacteria, and fungi and leads to death within the first two years of life unless treated by stem cell transplant (68). Bosma, Custer (69) observed mice of ty -65the C.B-17 strain that had impaired lymphopoiesis, caused by an autosomal recessive mutation (*scid*). These mice became the first mouse model of SCID, with mice homozygous for this recessive gene showing hypogammaglobulinemia and deficiency in functional T and B lymphocytes (69).

In 1988, McCune reasoned that if human hematopoietic stem cells (HSCs) and human thymus were introduced together into a mouse unable to reject them, T cell development and maturation could proceed in a fashion mimicking human physiology (11). McCune implanted C.B17 *scid/scid* mice (SCID mice) with human fetal thymus and injected them with human fetal liver cells, either intravenously or intrathymically (11). Mice were sub-lethally irradiated to ensure full reconstitution, as was seen previously in SCID mice implanted with long-term bone marrow cultures (70). These so-called SCID-hu mice showed human T cells in peripheral circulation at 6-7 weeks post-transplantation, but this population was no longer seen by 10-12 weeks (11). They also identified that the intravenously injected fetal liver cells could home to the implanted fetal thymus, and after 10 weeks, can be found in the peripheral circulation. If the thymus is engrafted in SCID mice alone, it eventually recedes over time. Therefore, a source of human progenitor cells needs to be provided (11, 71). Human fetal liver, in particular, is used as it is the primary site of hematopoiesis in humans between 8 to 24 weeks of gestation. The human fetal liver also contains progenitors for lymphoid, myeloid, and erythroid lineages (72) but limited numbers of mature CD3+ T cells (73). This reduces the risk of graft-versus-host disease (12) and allows human immune cells to engraft more efficiently than an adult or post-natal tissue (74).

Shortly after, Namikawa, Weilbaecher (12) implanted fetal thymus and liver concurrently. The resulting Thy/Liv mice show prolonged reconstitution with human immune cells with minimal graft-versus-host disease. These mice showed increased longevity in lymphopoiesis, even up to 15 months post-transplantation.

The SCID-hu mouse provided us with the first mouse model that could be used to study HIV-1 pathogenesis (75, 76). The first infection of a humanized mouse model (SCID-hu) with HIV-1_JRCSF_ was in 1988, shortly after McCune had developed the model (76). McCune, Namikawa (77) utilized this model to confirm, for the first time, the efficacy of the nucleoside analog azidothymidine (AZT) as an ART for HIV-1.

While providing a great stride forward in using humanized mice in HIV cure research, SCID-hu mice had some limitations. While the rapid generation of human thymocytes and naïve T cells was possible, mature T cells are primarily restricted to the implanted thy/liver organoid. Furthermore, these mice do not produce functional immune responses that recapitulate the human immune response.

### Peripheral Blood Leukocyte (Hu-PBL) Mouse

Mosier and colleagues conducted the first iteration of humanizing SCID mice by transferring human PBMCs in 1988 (13). These mice were susceptible to HIV-1 infection, with 50% of them presenting with detectable viral RNA 16 weeks post-infection (78). Infection of hu-PBL-SCID mice as early as 2 hours post reconstitution has led to productive HIV-1 infection and a dramatic decrease in CD4+ T cell numbers (79).

The establishment of non-obese diabetic (NOD) mice further progressed the field of humanized mice (80, 81). These NOD mice showed defects in the innate immune system, resulting in better engraftment when crossed with SCID mice. Further development came from crossing these so-called NOD-SCID mice with those who had the IL-2Rγ-chain null mutation (82) or truncation of the intracellular signaling domain (83). This common cytokine receptor gamma chain is required for the signaling of IL-2, IL-4. IL-7, IL-9 IL-15, and IL-21 (84–90) and its deletion completely abrogates murine Natural Killer (NK) cell development and function (82) which has shown to negatively impact engraftment of human lymphoid cells in mice (91). Modern versions of hu-PBL mice utilize NOD/SCID/IL2rγ-null (NSG) or BALB/c-Rag2null IL-2Rγnull (BRG) backgrounds as they provide better reconstitution (14–16). These mice show partial functionality (13, 78, 92, 93) but lack *de novo* multilineage hematopoiesis and therefore are absent of a primary immune response. They are also amenable to HIV-1 infection (14, 94, 95) and are responsive to HAART (95). This model is particularly suited to studying GVHD (16, 96, 97), simultaneously creating a significant limitation in this model’s utility for long-term HIV-1 studies.

### Hematopoietic Stem Cell (HSC) Mouse

Following the early success of reconstituting human immune systems in SCID mice, Kamel-Reid and Dick (17) intravenously infused hematopoietic stem cells from human bone marrow into the SCID mouse as well as the bg/nu/xid mouse, which are deficient in natural killer responses. They found bg/nu/xid mice show higher levels of human progenitors and, in addition to T and B cells, found that macrophage progenitors can be isolated and cultured *in vitro* (17). It was subsequently identified that by providing these HSC humanized mice with erythropoietin, human mast cell growth factor and human IL-3 stimulated the immature cells from human bone marrow to differentiate into cells of the erythroid, myeloid, and lymphoid lineages (28).

The cell surface sialomucin-like adhesion molecule CD34 is widely accepted as a marker for human HSCs (98, 99), having showing both short-term (98) and long-term (100) colony-forming potential *in vitro* and allowing the differentiation of both myeloid and lymphoid cell lineages in NOD/SCID mice (101). Péault, Weissman (18) isolated CD34+ cells from human bone marrow and human fetal liver and was able to reconstitute human fetal thymus *in vitro* and then implanted them into SCID mice. The third source of HSCs is umbilical cord blood (UCB). High levels of HSCs can be isolated from fetal liver, bone marrow, and UCB, although they produce varying levels of lymphoid, myeloid, and erythroid progeny (102). Hao, Shah (103) showed that CD34+CD38- cells isolated from UCB have a higher cloning efficiency, proliferate more rapidly in response to cytokine stimulation, and generate more progeny than those derived from human bone marrow. Furthermore, as few as 500 CD34+CD38- cells separated from UCB were able to reconstitute NOD/SCID mice (104).

Several mouse backgrounds have been utilized for reconstitution with HSCs, including NOD/SCID (104), NOD/Shi-Scid mice (105). NOD/Shi-*scid/IL2R*γ*null* (NOG) (106) and NSG (19, 107). McDermott et al. performed a comparative analysis of each mouse background (108). They used the current optimized methods for each mouse model, which included interfemoral injection of HSCs to all mice and anti-CD122 (IL-2Rβ) treatment for NOD/Lt-*scid* and NOD/Shi-*scid*, to inhibit NK cell activity (109). They identified that NSG and NOG mice had superior engraftment in the thymus and spleen compared to NOD/Lt-*scid* and NOD/Shi-*scid* mice. Also, NSG mice showed 1.5-fold greater engraftment in the bone marrow over NOG, NOD/Lt-*scid*, and NOD/Shi-*scid* mice (108). Finally, at limiting doses of HSCs, female NSGs showed improved engraftment compared to males (108).

Successful infection with HIV-1 through multiple routes have been demonstrated in CD34+ reconstituted NOG (110–114), BRG (115–117), NRG (118) and NSG mice (65, 119–124). They are also responsive to HAART (65, 118, 119) and have significant potential to contribute to our understanding of HIV-1 latency and therapeutic studies.

### DRAG Mouse

Impaired T cell development and consequent lack of antibody class switching in HSC humanized mice is mainly attributed to the lack of donor matched HLA molecules in the mouse thymus. Lack of a thymic environment hampers the negative selection of self-reactive T cells by which autoimmunity is prevented. Danner, Chaudhari (20) hypothesized that by expressing human HLA class II molecules in a transgenic mouse model, they could rescue the development of CD4+ T cells and consequently B cell development and antibody class switching. They generated NOD.Rag1KO.IL2RccKO mice expressing HLA-DR4 (0401), abbreviated DRAG, and at 4-6 weeks old intravenously injected them with CD34+ HSCs isolated from HLA-DR*0401 positive UCB (20). When compared against HLA mismatched recipients, higher levels of reconstitution of CD4 + T cells were seen in DRAG mice comparable to human blood levels. However, levels of CD8+ T cells did not show as drastic an increase. These mice showed the presence of dendritic cells (2.9% in spleen) and NK cells (0.05% in spleen), although their development appeared to be unaffected by HLA-DR4 expression. T cells isolated from DRAG mice showed vigorous responses, similar to that of PBMCs from healthy volunteers upon stimulation with either CD3/CD28 or PMA/ionomycin. B cell reconstitution was seen at similar levels to control mice, although IgM levels were significantly higher in DRAG mice. Interestingly, DRAG mice showed substantial IgG reconstitution, confirming that the mechanism of immunoglobulin class switching is rescued in DRAG mice, a feature lacking in most other humanized mouse models. Further, DRAG mice showed reconstituted plasma levels of all human IgG subclasses, with IgG2 being the most prevalent.

Studies have shown that DRAG mice are susceptible to HIV-1 infection, similar to other HSC reconstituted mice (125, 126). The replication-competent virus was isolated from plasma, lymph nodes, bone marrow, spleen, gut, brain, and female reproductive tissue upon a single intravaginal challenge of purified primary HIV-1 (125). High reconstitution in the gut and female reproductive tract, particularly with CXCR5+PD-1++ Follicular T helper cells, which are highly permissive to HIV-1 infection, are potentially a significant contributor to the above (126). Also, plasma viral loads were stable as far as 18 weeks post-infection, making the DRAG mouse a suitable model system, particularly for long-term vaccination studies.

### Bone Marrow-Liver-Thymus (BLT) Mouse

While implantation of Thy/Liv into SCID or NOD/SCID mice results in thymopoiesis and functional T cells, strong *in vivo* immune responses are not observed. In 2006 (21), Lan et al. combined the implantation of fetal Thy/Liv with the simultaneous transplantation of CD34+ fetal liver cells in NOD/SCID mice (21). In the same year, Melkus, Estes (22) performed a similar transplant on the NOD-SCID background, coining the term ‘BLT’ to describe these bone marrow-liver-thymus humanized mice (22). A significant advantage of the BLT model is that it allows the development of MHC-restricted T cells due to the presence of an autologous human thymic environment (22). This iteration of the humanized mouse showed stronger *in vivo* immune responses and repopulates with multiple lineages of immune cells, including T cells, B cells, NK cells, Dendritic Cells, Neutrophils, and Monocytes distributed through multiple organs, including bone marrow, lymph nodes, spleen, thymus, liver, lung, digestive and reproductive tracts (21–23, 42, 43, 46, 127, 128).

Soon after its development, Sun, Denton (128) identified that BLTs contain CD4+ T cells throughout the gut-associated lymphoid tissue (GALT), including the colon and rectum, and thus hypothesized that they would be ideal for modeling intrarectal transmission, a predominant form of HIV-1 transmission. BLT mice were inoculated intrarectally with a single dose of cell-free HIV-1 (LAI CXCR4 strain), and six out of seven infected BLTs were found positive for viral RNA and the presence of p24 antigen (128). Similarly, Denton, Estes (127) showed that BLTs were susceptible to vaginal transmission of HIV-1 (CCR5-tropic JR-CSF). Infection was prevented in BLTs pre-treated with a daily dose of the ARTs emtricitabine and tenofovir diosoproxil fumarate (FTC/TDF), making the BLT model additionally suited for preclinical testing of pre-exposure prophylactics (PrEP). Subsequent studies of topically administered 1% tenofovir in BLT mice also showed partial protection against vaginal transmission of HIV-1 (129).

Brainard, Seung (23) infected BLT mice made with mice of both NOD/SCID and NSG backgrounds, implanting tissue under both right and left kidney capsules. Stoddart, Maidji (24) also demonstrated that NSG reconstitution was highly superior to NOD/SCID reconstitution (24). However, Denton, Nochi (42) showed higher levels of intraepithelial lymphocytes in the small and large intestines of NOD/SCID BLTs.

The functional human cellular immune response observed in BLTs makes it a particularly valuable model for studying HIV specific immunity. HIV infection in BLT humanized mice is associated with both CD8+ T cell activation (23, 130, 131) as well as HIV-specific IgM and IgG (23, 40). HIV-1 specific CD8+ T cell responses show strong similarities to human cellular immunity and result in rapid viral escape in BLT mice (131). Furthermore, mice created with tissue from elite controllers that express the HLA-B*57 allele exhibited enhanced Gag mediated control of viremia (131).

While substantial reconstitution with B cells is observed in BLT mice, they are considered primarily immature, and antibody class-switching is thought to be defective. Interestingly, it has been speculated that the humoral immunity observed in BLT mice in response to viral infection is driven by extra-follicular or ‘innate-like’ B cells rather than conventional post-germinal B cells (40, 132, 133). This inadequate antibody response has led to the proposal of the BLT mouse as a model for hypogammaglobulinemia (134).

Crucial to HIV-1 therapeutic studies is the establishment of latency in the BLT mouse model. Denton, Olesen (135) used a commonly used combination ART regimen, a combination of tenofovir, emtricitabine, and raltegravir, to suppress viral replication in BLT humanized mice, and thereafter isolated CD4+ T cells and cultured them ex vivo. Stimulation of these CD4+ T cells with Phytohemagglutinin and IL-2 led to a rebound in viral load (135).

### Triple Knockout (TKO)-BLT

While modern BLT and humanized mouse models primarily use NOD/SCID backgrounds, there is the potential benefit of developing a BLT model on the C57BL/6 background due to the wide availability of transgenes and genes inactivation as well as its relative resistance to radiation. A significant barrier to this is the expression of a different form of the signal recognition protein α (SIRPα) receptor by C57BL/6 mice, which does not recognize the human ligand CD47 (integrin associated protein) unlike in NOD mice (136, 137). Recognition of CD47 on transplanted human cells by SIRPα on mouse macrophages leads to the transmission of inhibitory signals preventing their phagocytosis (138, 139). The Hasenkrug lab demonstrated that creating C57BL/6 Rag2-/-γc-/- (TKO) facilitated a CD47-negative environment, which leads to tolerizing of cells without the need for the SIRPα-CD47 interaction, thereby allowing successful long-term human immune systems in TKO-BLT mice (25, 26).

The TKO-BLT mice showed no clinical signs of graft-versus-host disease (GVHD) up to 29 weeks post-transplantation compared to NSG-BLT mice, of which a third had died from lethal GVHD at the same timepoint (26). Furthermore, TKO-BLT mice developed human GALT, including CD4+ T cells, supporting mucosal HIV infection through intraperitoneal and intrarectal routes (26). HIV-1 infected TKO-BLTs showed HIV gp120_JR-CSF_ specific IgG antibodies demonstrating both antibody responses and T-cell dependent class switching (26). Furthermore, cells from the spleens of HIV infected TKO-BLTs were assayed by IFN-γ ELISPOT and were shown to be responsive to a broad range of peptide pools spanning the HIV-1_JR-CSF_ proteome (26). Similar to NSG-BLT mice, the TKO-BLT can also recapitulate HIV-1 latency upon treatment with ART up to 18 weeks, with viral rebound seen upon removal of therapy (27). GVHD is a limitation in the BLT humanized model (140). Resistance to GVHD makes the TKO-BLT particularly suited to long-term studies required to study HIV-1 latency and measure the lasting efficacy of current curative approaches.

## Humanized Mice in HIV-1 Cure Research

The primary barrier to eradicating HIV-1 is its integration into the host genome and continued persistence in a non-replicating or ‘latent’ state, even under ART (141–143). Furthermore, chronic infection with HIV-1 leads to significant disruptions in the host’s immune response (144). Consequently, latently infected cells are difficult to find, and the host immune response is inefficient in killing these cells. Therefore, innovative strategies are required to find, measure, and ultimately eradicate the latent HIV-1 reservoir.

Two definitions exist for a cure for HIV. The complete eradication of the virus from all cells in the body is defined as a sterilizing cure. In contrast, a functional cure would be a treatment that would lead to HIV+ people being able to halt ARV treatment while maintaining long-term viral suppression and preventing transmission of the virus. The humanized mouse models described in this review have played a vital role in pre-clinical testing of many approaches to an HIV-1 cure, with some directly leading to clinical trials.

### Latency Reversal

One major strategy in the fight for a sterilizing cure is the “shock” and “kill” approach. This strategy involves utilizing latency reversal agents to ‘shock’ the latently infected cells into resuming replication of the virus, allowing the immune system to recognize and kill infected cells or for virus-induced cytolysis to occur. Several classes of LRAs have been identified which include histone deacetylase inhibitors (HDACis) (145–147), histone methyltransferase (HMT) inhibitors (148), DNA methyltransferase inhibitors, Protein Kinase C (PKC) activators (149), bromodomain inhibitors (150), Disulfiram (a drug used to treat chronic alcoholism) (151), agonists to Toll-like receptor 7 (152) and cytokines such as IL-15 (153).

One of the most well-studied classes of LRAs, HDACi’s work by inhibiting the enzymes that remove acetyl groups from histones, leaving them in an open state, and increasing the ability for transcription to resume. While HDAC inhibitors such as Panobinostat successfully increase levels of histone acetylation *in vivo* (154) and reactivation of latently infected cells *ex vivo* (149, 155), reactivation of HIV-1 replication was not seen in ART-treated HIV-1 infected BLT mice (154).

Other classes of LRAs impact cellular factors such as NF-κB and pTEFB by either increasing their expression or activating them. Protein kinase C (PKC) agonists are one such class that activates the NF-κB pathway leading to reactivation of latent HIV-1 (156, 157). Prostratin, a PKC agonist, was one of the earliest LRAs of this class identified with potency in reactivating latent HIV-1 (158). Additionally, IDB and Bryostatin 1 can effectively reactivate viruses in cells isolated from HIV-infected patients (149, 159). Marsden, Loy (160) identified a synthetic analog of the PKC modulator bryostatin 1, SUW133, which when intraperitoneally injected into ART-treated HIV-1 infected BLT mice, reactivated HIV-1 more potently, was more tolerable than the natural analog, and led to infected cells’ death.

Bobardt, Kuo (161) showed that an inhibitor apoptosis protein antagonist (IAPa) reactivates latent HIV-1 by degrading the ubiquitin ligase BIRC2, a repressor of the non-canonical NF-κB pathway. Substantial reactivation was seen *ex vivo* in latently infected CD4+ T cells isolated from ART-treated HIV-1 infected BLT mice at much greater levels than the HDACis panobinostat and vorinostat as well as another IAPa LCL-161 (161). Similar results were seen *in vivo* with AZD5582, which also works as a SMAC mimetic and activates the non-canonical NF-kB pathway (162). Upon a single dose of 3mgkg-1 of AZD5582 HIV-1 RNA was detected in 3 out of 6 and 3 out of 4 ART-treated HIV-1 infected BLT mice, with no signs of general toxicity (162).

Specific cytokines have dual potential to both reactivate HIV-1 replication and simultaneously activate host immune cells such as NK cells to kill infected cells (153, 163, 164). Recently, the IL-15 ‘super-agonist’ N-803 tested in the BLT humanized model showed reactivation of the viral reservoir only upon depletion of CD8+ T cells, showing further nuance in the interactions between LRAs, viral reactivation, and immune cells (165).

Interestingly, Llewellyn, Alvarez-Carbonell (166) found that HSC mice (NSG fetal liver CD34+) reconstitute with human microglial cells in the brain when endogenous microglia are depleted using the chemotherapeutic agent busulfan at much greater levels than mice pretreated with irradiation. These human-derived microglial cells could be infected with HIV-1 and, upon exposure to the MAO inhibitor phenelzine, they could reactivate latent virus *ex vivo* (166). These results further emphasize the potential for humanized mouse models in studying latency reversal in multiple tissue reservoirs of HIV-1.

Unfortunately, while many LRAs have successfully reactivated HIV, the subsequent killing by viral cytopathic effects, killing by host immune cells, or intensifying ART has proven ineffective in the clinic (167). This is partly due to dysfunctional CTLs (168) and NK cells (169–171) and due to the presence of escape mutant viruses in the latent reservoir (172). Therefore, to enhance the ‘kill’ arm, multiple immunotherapies, gene therapies, and pharmacological interventions have been explored.

### Broadly Neutralizing Antibodies (bNAbs)

The HIV-1 envelope protein (env) is a trimeric glycoprotein of gp41 and gp120 heterodimers and is on the virus’s surface and consists of multiple sites that can be targeted by host antibodies. The majority of neutralizing antibodies that develop in humans are strain-specific, but a small subset of antibodies can bind to and neutralize a broad range of HIV-1 strains (173–176). Initial attempts to utilized these broadly neutralizing antibodies (bNAbs) in HIV-1 therapy proved unsuccessful in both humanize mice (47) as well as in the clinic (177, 178). However, with the advent of single-cell antibody cloning methods, highly potent bNAbs have been identified (179). Over the past decade, humanized mice have proved instrumental in developing therapies utilizing bNAbs for viral suppression and eradicating latently infected cells.

Klein, Halper-Stromberg (48) showed that treatment with a combination of bNAbs (45-46^G54W^, PG16, PGT128, 10-1074, and 3BC176) that target different epitopes could suppress viral replication in HIV-1 infected hu-HSC mice. These humanized mice were then infected with HIV-1_YU2_, and treated with a penta-mix treatment therapy reducing viral loads to undetectable levels for up to 60 days post removal of therapy compared to only 10 days when treated with ART. Shortly after, Horwitz, Halper-Stromberg (49) showed in the same mouse model that lowering the viral load by ART followed by treatment with bNAb monotherapy (3BNC117, PG16, or 10-1074) can similarly lead to control of viremia until antibody titers dropped to low or undetectable levels. Also, a single injection of adeno-associated virus directing expression of a single bNAb suppressed viral replication after the termination of ART (49). Following these studies, Halper-Stromberg, Lu (50) hypothesized that these capabilities of bNAbs could be harnessed to amplify the “kill” arm with bNAb therapy after reactivation of the latent reservoir. They used three LRAs; the HDACi Vorinostat, the BET protein inhibitor I-BET151, and CTLA, a T cell inhibitory pathway blocker given individually or in combination (50). They discovered that combination treatment with the three LRAs with the trimix bNAb treatment (3BNC117, PG16, or 10-1074) in humanized mice reduced the viral reservoir, evidenced by reduced viral rebound at significantly higher levels than with single LRAs (50). They further showed that bNAbs exert this effect primarily through Fc mediated functions by comparing their bNAbs to those with mutated Fc regions (50).

These results provided the preclinical evidence that combination bNAb therapy is a critical tool that can be utilized in HIV-1 eradication and has since led to the translation of these ideas to the clinic (180). In an open-label clinical trial, Caskey, Klein (181) showed that monotherapy with a single 30mgkg^-1^ infusion of 3BNC117, the antibody targeting the CD4 binding site, was able to suppress viral load in HIV-1 infected individuals for up to 28 days. Also, in the following phase II trial, the same therapy suppressed HIV-1 viral load in infected individuals on average for 10 weeks, upon four doses given two weeks apart (182). Further investigation by adoptive transfer of patient T cells into NRG mice identified that these effects are not limited to preventing new infection of cells, but also aids in the clearance of infected cells by Fcγ receptor-mediated engagement (183). Shortly after, Caskey, Schoofs (184) tested 10-1074, which targets a glycan on the V3 loop of the HIV-1 envelope spike, as monotherapy in HIV-1 infected patients. Similarly, at a dose of 30mgkg^-1,^ 11 out of 13 HIV-1 positive individuals showed suppression of viral loads. The two that failed to respond carried mutations leading to single-amino-acid changes, making them 10-1074 resistant before treatment (184). Unfortunately, while monotherapy with bNAbs was proven to be safe and efficacious, in both cases, it led to viral escape (181, 183, 184).

In contrast to monotherapy, but similar to the results in humanized mice, combination therapy with 3BNC117 and 10-1074 at a dose of 30mgkg^-^ was found to be both safe and more effective at viral suppression in humans (185–187). When administered upon treatment interruption three times at three weeks apart, 11 HIV-1 infected individuals resulted in viral suppression for a median of 21 weeks post final dose of antibody therapy (186). Furthermore, none of the rebounding viruses showed resistance to both therapeutic bNAbs. Similar to data generated in humanized mice, these data demonstrate the enhanced utility of combination bNAb therapy to suppress viral replication (186). Moreover, combination bNAb therapy during treatment interruption led to increases in antigen-specific CD8+ T cells that expressed IFN-γ. TNF-α, MIP1-β, and CD107a in all individuals at 6-7 weeks while plasma bNAb levels were at their peak (188). While latency reversal agents combined with bNAB therapy have yet to be tested in the clinic, the evidence provided so far shows a clear line from preclinical experiments in humanized mice to the successful translation of these therapies into the clinic.

### Chimeric Antigen Receptor (CAR) T Cell Immunotherapy

Since the advent of CAR T cell therapies and their successful use in treating B cell malignancies (189–191), several approaches have been taken to utilize this technology to target HIV-1 infected cells.

Kitchen, Bennett (53) transduced HSCs isolated from human fetal liver with an HLA-A*0201 restricted T cell receptor (TCR) targeted towards the HIV-1 gag SL9 epitope and injected them into human thymic implants in SCID-hu mice, providing them with an optimal environment for development. This led to the development of anti-HIV TCR+ CD8+ T cells, which produced Interferon-γ (IFN- γ) and lyse target cells in response to SL9 peptide stimulation *ex vivo* when biopsied from the thymus after seven weeks (53). In a subsequent study Kitchen, Levin (54) transduced HSCs with the same vector. They tested the ability of these anti-HIV TCR+ CTLs to suppress viral replication in a modified NSG-BLT model, which received a fetal thymus/liver implant along with the transduced HSCs. They found a significant decrease in plasma viremia at two weeks and six weeks and a higher percentage of CD4+ cells in mice transduced with the HIV-1 specific TCR when compared against a non-specific TCR control (54). Similar results were seen upon analyses of other organs of infected mice, with lower HIV-1 DNA at six weeks in the spleen, bone marrow, and human thymus (54). Importantly, their analysis of the viral RNA in the blood showed that within this time, no viral escape had occurred in response to the selective pressure of the SL9 specific TCR (54).

While modification with molecularly cloned anti-HIV TCRs showed to be effective, its application is restricted by HLA type, and several effective HIV CTLs use uncommon HLA alleles (192). A second alternative approach is a CD4ζ CAR, a chimeric molecule consisting of the extracellular and transmembrane domains of the human CD4 molecule fused to the signaling molecule of the CD3 ζ chain. Zhen, Kamata (55) transduced human HSPCs with an HIV-specific CD4ζ CAR and two antiviral genes (Triple CAR vector) and transferred them into NSGs transplanted with fetal thymus and liver (55). The antiviral genes included a small hairpin (sh) RNA molecule specific to human CCR5 and shRNA targeting specific HIV-1 long-terminal repeat (LTR) sequences to prevent the newly CD4 CAR-expressing cells from being infected by HIV-1, an obstacle previously observed with this approach (55). The Triple CAR construct was expressed on T cells, NK cells, B cells, and myeloid cells (55). They found these cells are resistant to HIV infection themselves, suppressed HIV replication *in vivo*, and isolated CD4ζ CAR-expressing cells could produce IFN- γ and tumor necrosis factor (TNF)-α when cultured with virally infected cells (55).

While CD4ζ CARs showed promise *in vitro* and in preclinical studies, clinical trials showed that while they survive for at least 11 years post-transfusion, they could not suppress the viral reservoir in a sustained manner (193). Therefore, Leibman, Richardson (56) decided to identify several components of the CD4ζ CAR construct to be optimized to improve their antiviral function. First, they switched from a murine retroviral vector (MMLV) to a lentiviral vector, as MMLV targets promoter regions while lentiviral vectors preferentially integrate into open reading frames (56). Next, they switched from the PGK promoter to the EF1α promotor, which induces higher expression better sustained as T cells reach a resting state (56). Thirdly, they swapped the CD4 transmembrane domain to a CD8a transmembrane domain to promote CAR dimerization (56). Finally, they assessed the inclusion of different co-stimulatory receptors such as 4-1BB and CD28 (56). While each modification individually produced significant improvements to target cell killing *in vitro*, in combination, they resulted in a 50-fold increase in potency over the original construct (56). To test their optimized CD4ζ CARs *in vivo*, they utilized a modified hu-PBL mouse as an HIV-1 treatment model. Briefly, they transferred CD8+ T cell-depleted PBMCs into NSGs, transfused them with HIV-1 infected CD4+ T cells, treated with 200mg/kg TDF for four days, and after removal of ART, transfused the mice with their CD4ζ CAR transduced CD8+ T cells (56). They found that mice infused with the optimized CD4ζ CARs T cells controlled HIV-1 replication better and expanded to greater levels *in vivo* than their first-generation counterparts (56).

In addition to CD4ζ, CARs with a single-chain variable fragment (scFv) derived from bNabs have been utilized. However recent clinical trials using bNAb monotherapy led to viral rebound upon interruption of ART (181, 184, 194). Bardhi, Wu (57) showed that a hexavalent fusion protein which consisted of m36.4, the scFv heavy chain only domain which targets the gp120 co-receptor binding site and mD1.22 an engineered mutant of the CD4 extracellular domain when intravenously injected into a modified hu-PBL model induced NK cell-mediated killing of HIV-1 infected cells. The model they used involved injecting human PBMCs intrasplenically (hu-spl-PBMC-NSG) into NSG mice followed by intrasplenic inoculation with HIV-1 (57, 58, 164). Subsequently, Anthony-Gonda, Bardhi (58) utilized m36.4, mD1.22, and a fusion inhibitor peptide C46, individually or in combination, to develop novel multi-specific CAR constructs. Using their hu-spl-PBMC-NSG model, they discovered that their duoCAR construct, by which m136.4 and mD1.22 were expressed as individual CARs on the same cell, was able to suppress HIV-1 infection for up to 30 days while mitigating CD4+ T cell depletion, compared to their monoCAR or untransduced counterparts (58). Altogether, these findings clearly show the utility of various humanized mouse models in elucidating CAR-based immunotherapy *in vivo* efficacy in the clearance of HIV-1 infected cells.

### Gene Editing With Designer Nucleases

The first patient cured of HIV to date was treated for myeloid leukemia with total body irradiation and two allogeneic hematopoietic stem-cell transplants (HSCT) from donors with the CCR5Δ32/Δ32 mutations (195). While the second patient exhibiting HIV-1 remission reported in 2019 received no radiation and only one HSCT transplant (196). These treatment methods are not feasible for the wide-scale treatment of otherwise healthy HIV-positive people. However, these successes have inspired the gene-editing field to target the CCR5 gene to induce resistance to HIV-1 infection (at least through CCR5 tropic HIV-1).

To date, three major types of nucleases are used for genome editing, namely, zinc finger nucleases (ZFN), transcription activator-like effector nucleases (TALEN), and the Cas9 endonuclease with clustered regularly interspaced short palindromic repeats (CRISPR). All three utilize a similar overall mechanism of binding a target DNA sequence, creating a double-stranded break allowing the DNA to undergo repair through the error-prone non-homologous end joining (NHEJ), resulting in small insertions or deletions disrupting gene expression [reviewed in (197)].

ZFN consist of pairs of zinc finger DNA binding domains that recognize three base pairs of DNA and are covalently linked to complementary halves of a FokI restriction endonuclease. ZFNs have been utilized to confer resistance to HIV-1 infection in resting CD4+ T cells through the disruption of the CCR5 gene in humanized mouse models (59, 60). Similarly, Holt, Wang (123) showed human HSCs in which CCR5 was disrupted by ZFN, were able to engraft in NSG mice successfully and led to the development of CCR5-tropic HIV-1 resistant cells. Also, ZFNs targeting the CXCR4 co-receptor on CD4+ T cells have shown success in protecting from HIV-1 infection in humanized mice, although resistance was eventually lost upon selection for CCR5-tropic mutants (61, 62). Following these promising results in humanized mice, the first phase 1 human clinical trial with ZFN-CCR5 mutated CD4+ T cells was conducted, showing that these genetically modified CD4+ T cells were more resistant to HIV-1 infection and led to decrease viral loads during ART interruption (198).

The gene-editing field was revolutionized by the discovery of CRISPR/Cas9 in prokaryotes (199, 200) and its role in bacterial and archeal adaptive immunity against invading viruses (201). Unlike ZFNs, CRISPR/Cas9 relies on short sequences of guide RNA (gRNA) to target the Cas9 nuclease to the complementary DNA sequence to be edited. This feature of CRISPR/Cas9 makes it more sequence-specific and easier to design and produce, leading to its first use in gene editing by Jinek, Chylinski (202), a landmark discovery leading to a Nobel prize award in chemistry in 2020. Not long after, CRISPR/Cas9 was used to target the HIV-1 LTR in human cell lines resulting in a significant decrease in HIV-1 expression upon stimulation (203). Recently, CRISPR with *Staphylococcus aureus* Cas9 (SaCas9) delivered by a lentiviral vector improved primary CD4+ T cell resistance to HIV-1 infection. Also, transplanting these cells into NCG humanized mice resulted in enhanced survival upon challenge with CCR5 tropic HIV-1_YU2_ (64).

In addition to targeting host factors, another gene-editing approach that has picked up steam is to directly target the integrated HIV-1 provirus for excision (203–205). This approach has been used to remove the entire HIV-1 genome between the 5’ and 3 LTRs from latently infected human CD4+ T cells (206). Humanized mice have shown to be invaluable in testing these approaches in a model that accurately recapitulates HIV latency, as is seen in humans. Yin, Zhang (66) discovered that *in vivo* excision of the HIV-1 provirus with a SaCas9 with multiplex single-guide RNAs (sgRNA) was successful in a small cohort of BLT mice. A TALEN targeting the TATA-box of the HIV-1 LTR has also been used successfully to clear HIV-1 *ex vivo* on splenocytes derived from humanized mice (63).

Recently the Gendelmann lab combined long-acting slow-effective release (LASER) ART treatment with consequent CRISPR/Cas9 treatment to excise HIV DNA (65). They showed in 3 donor cohorts of hu-HSC mice, over a third 9/23 of the mice showed no rebound of the infectious virus following combination treatment than LASER ART or CRISPR/Cas9 treatment alone (65). Multiple organs, including the spleen, bone marrow, gut, brain, liver, kidney, and lung of these mice were assessed and showed no viral rebound (65). This data provides a solid foundation for the efficacy of these gene-editing approaches *in vivo* and can pave the way for human clinical trials.

### Block and Lock

While the therapeutic approaches discussed so far have focused on the complete eradication of HIV-1 reservoirs, the Valente lab took an alternate approach to lock HIV-1 in its latent state by targeting the HIV-1 Tat protein (67, 207). Tat is transcribed early in the HIV-1 lifecycle and recruits the necessary transcription factors to enhance viral transcription and stabilize elongation (208). Specifically, Tat binds the 5’ terminal region of the transactivation response element (TAR) (208) on HIV mRNA and recruits the positive transcription elongation factor B (PTEF-b) (209). PTEFb is composed of cyclin T1 and cyclin-dependent kinase 9 (CDK9), promoting transcription elongation from the viral promoter (210). Also, Tat has shown to recruit chromatin remodeling factors such as SWI/SNF (211, 212) and histone acetyltransferases (213–215) allowing chromatin to remain in an open state and thus allowing easier access to transcription factors such as nuclear factor-kB (NFkB).

Targeting viral factors such as Tat is a major focus of antiretroviral therapy research, mainly due to the absence of a host cellular homolog, resulting in less toxicity. The Valente Lab discovered that didehydro-cortistatin A (dCA), a synthetic analog of the natural product cortistatin A, potently inhibits Tat-dependent transcription activity in both acutely and chronically infected cells (216). dCA was also found to act additively with conventional HAART, inhibiting spontaneous viral particle release from CD4+ T cells in virally suppressed subjects (216). Soon after that, they showed that dCA effectively inhibits viral reactivation by a PKC agonist or by antigenic stimulation of primary latently infected cells isolated from ART-treated individuals (207). Furthermore, they demonstrated that dCA, unlike conventional ART, can reduce the base level cell-associated HIV-1 RNA production by decreasing RNA Polymerase II recruitment to the viral promoter (207). Primary CD4+ T cell cultures alone cannot fully capture the characteristics of latently reservoirs, as these use clonal HIV strains and specific cytokine cocktails to prolong lifespan. These conditions may transform these cells and alter cell-subset representation. Therefore, in a subsequent study, Kessing, Nixon (67) tested the ability of dCA to suppress viral reactivation in a BLT mouse model. Humanized mice were infected with HIV-1_JRCSF_ and treated with ART for three weeks, at which point dCA was combined with ART for the fourth week (67). Strikingly, upon treatment interruption, viral rebound was delayed up to 19 days in mice treated with dCA compared to the controls, which showed rebound between 3-7 days upon treatment interruption (67). Unfortunately, certain viruses develop resistance to dCA through a combination of mutations in Nef and Vpr that increase NF-κB activity and lead to a higher Tat-independent basal transcription (217). The discovery of additional transcriptional inhibitors that promote deep latency through different mechanisms, used in combination with dCA could be the way forward for the block and lock strategy. In *vivo* analysis in humanized mouse models will be critical in their translation to the clinic.

## Limitations of Humanized Mice

Despite the fact that they are an advantageous small animal model for HIV-1 cure research, humanized mice have their limitations. Hu-PBL mice and, to a lesser extent, hu-HSC and BLT mice occasionally develop graft-versus-host disease. GvHD is characterized by lymphocytic infiltration, progressive inflammation, and sclerosis in multiple organs, eventually leading to death (140). Moreover, the associated excessive inflammation can confound results from long-term HIV-1 studies. The use of NSG mice over NOD/SCID mice for the construction of BLT humanized mice was found to delay the onset of GVHD, although it did not decrease its incidence (140). Furthermore, even with many advances over the years, here remain deficiencies in the immune response of humanized mice compared to a complete human immune system. First, they lack robust humoral immune responses due to limited class switch recombination and mutation rates, limiting the study of B cell responses, particularly in vaccination studies (23, 134). Also, hu-PBL, and hu-HSC mice lack human HLA restriction of T cells, leading to deficiencies in the development and functionality of T cells, a significant advantage of the BLT model.

On the other hand, the construction of BLT mice requires significant technical expertise to produce, and access to fetal tissue for scientific research continues to become scarce. Moreover, the murine environment lacks human cytokines and poor cross-reactivity with murine cytokines leads to incomplete development of some human myeloid and lymphoid cell subsets. Several solutions for this include treatment with exogenous human cytokines. For instance, periodic intraperitoneal injection of human IL-15/IL-15Rα is used to support the proliferation and survival of NK cells (46, 218). Other strategies involve knock-in and transgenic human cytokine insertions into immunodeficient mice. For example, human IL-2 and IL-15 transgenics (219), IL-15 and SIRPα knock-in (39), and IL-7 and IL-15 knock-in mice (220) promote NK cell development in humanized mice. Also, mice that comprise multiples gene modifications to produce the cytokines macrophage-colony stimulating factor (M-CSF), IL-3, granulocyte-colony stimulating factor (GM-CSF), and thrombopoietin have been established to support monocytes and macrophage development (33). While these modified humanized mouse models show potential in producing a more comprehensive innate immune system, their utility in HIV-1 persistence and therapeutic studies has yet to be evaluated.

Another limitation to long term persistence studies is the limited lifespan of mice (221), making studies lasting several years impractical. In these cases, an NHP model would be more suitable. Even though small animals are easier to house and maintain, they bring the additional constraint of having lower blood volumes and cell numbers for analyses, making *ex vivo* assays technically challenging to perform. Furthermore, the murine basal metabolic rate is different from that of humans and needs to be considered, particularly when dealing with pharmacological approaches (222). Lastly, the murine gut microbiome differs significantly from that of humans. This important topic has been reviewed in detail elsewhere (223, 224). The microbiome has several implications in HIV-1 infection, pathogenesis, and the host immune response [reviewed in (225–228)], and the microbiomes of inbred mice in a controlled environment aren’t capable of fully recapitulating these effects. To skew the microbiome to resemble that seen in humans, Daharsh, Zhang (229) treated BLT mice with broad-spectrum antibiotics to deplete the murine gut microbiome, followed by fecal transplants from healthy human donor samples *via* oral gavage. While this approach shifted the murine microbiome towards the corresponding human donors, they didn’t wholly resemble a human gut microbiome. Future developments may improve this premise, leading to a more comprehensive mouse model mimicking both a human immune system and the human gut microbiome.

## Conclusion

Regardless of its limitations, the humanized mouse is currently the only small animal model for HIV-1 and has proved to be an incredible resource in many facets of HIV-1 research in the past four decades. Large cohorts of mice can be generated from tissues derived from a single human donor, resulting in animals with genetically identical human immune cells. This approach allows us to control genetic variables and perform specific experiments such as adoptive transfers of lymphocytes, which would not be possible in most NHPs. Furthermore, humanized mice do not require a surrogate virus for infection and are susceptible to multiple routes of HIV-1 transmission. Moreover, tissue reservoirs harboring latent viruses can be analyzed, allowing a more straightforward comparison of the systemic effects of administered therapeutics, which is exceedingly essential for HIV-1 eradication efforts. Humanized mice have been critical in HIV-1 research, and we expect they will continue to play a significant role in ultimately translating a cure from bench to bedside.

## Author Contributions

SA and SP conceptualized the content of and wrote the article. All authors contributed to the article and approved the submitted version.

## Funding

This work was supported by NIH RO1 AI116282 (SP), and unrestricted funds from The Scripps Research Institute, La Jolla, CA (SP).

## Conflict of Interest

The authors declare that the research was conducted in the absence of any commercial or financial relationships that could be construed as a potential conflict of interest.
