# Corrigendum: Humanized Mice for the Evaluation of Novel HIV-1 Therapies

**DOI:** 10.3389/fimmu.2021.808068

**Published:** 2021-12-06

**Authors:** Shawn Abeynaike, Silke Paust

**Affiliations:** ^1^ Department of Immunology and Microbiology, The Scripps Research Institute, La Jolla, CA, United States; ^2^ The Skaggs Graduate Program in Chemical and Biological Sciences, The Scripps Research Institute, La Jolla, CA, United States

**Keywords:** humanized mice, BLT, DRAG, HIV-1 infection, viral latency, latency reversal, immunotherapy, gene therapy

In the original article, there was a mistake in **Table 1** as published. The authors incorrectly categorized thy/liv implanted SCID-hu mice as showing no multilineage hematopoeisis. To clarify, Namikawa 1990, showed that SCID mice implanted with both Thy/Liv displayed multilineage hematopoiesis. Specifically, they showed in addition to T cells (CD3, CD4 and CD8), the presence of mature and immature forms of myelomonocytic cells which stained positive for human CD15, as well as progenitors for erythroids and megakaryocytic lineages (1).

**Table 1:** Summary of humanized mouse models and their tissue-based chimerism.

	SCID-hu	hu-PBL	hu-HSC	BLT	TKO-BLT
**Mouse Model**	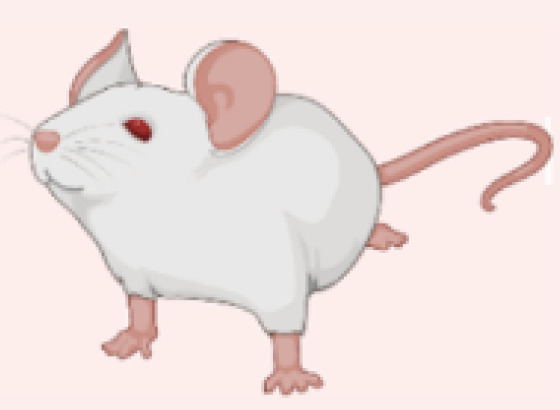	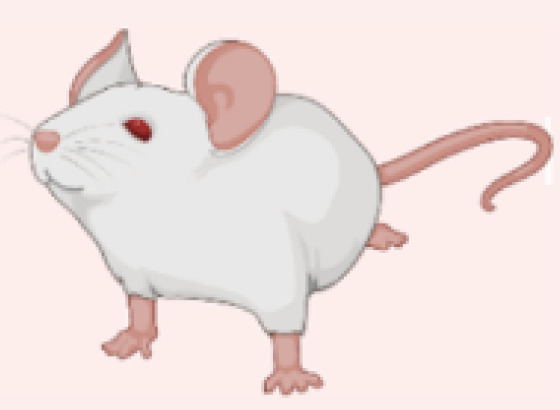	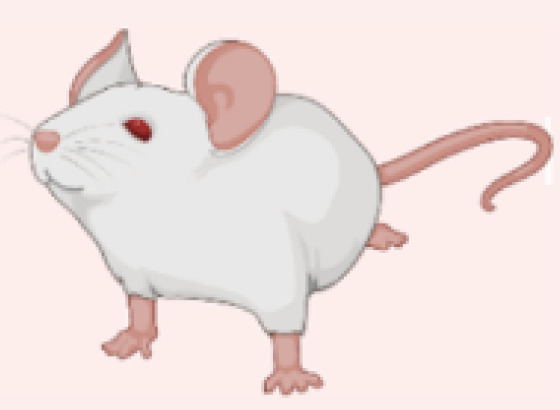	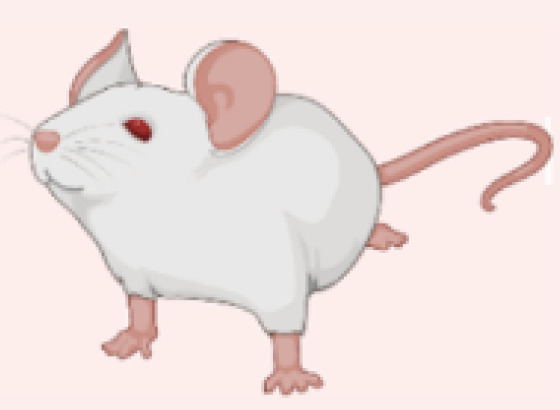	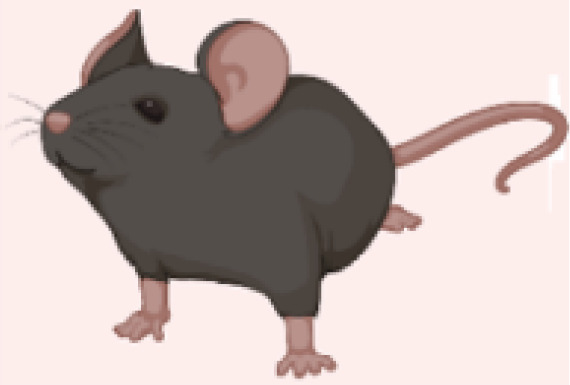
**Genetic Background**	C.B17scid/scid (SCID)	SCID NOD-SCID NSG BRG NCG	SCID NOD-SCID NSG BRG NRG DRAG	SCID NOD-SCID NSG NRG	C57BL/6 Rag2 ^-/-^ $g_{c}^{-/-}$
**Humanization Method**	Subcapsular Coimplantation of human fetal thymus and liver fragments	Intraperitoneal Injection of human PBMCs	Injection of CD34+ cells from cord blood/fetal liver	Coimplantation human fetal thy/liv with i.v. injection of CD34+ cells from fetal liver	Coimplantation human fetal thy/liv with i.v. injection of CD34+ cells from fetal liver
	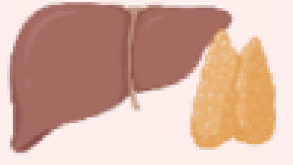	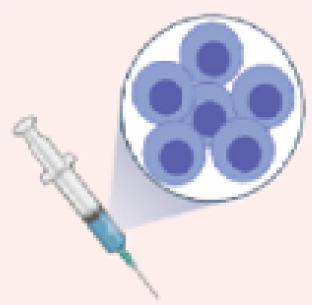	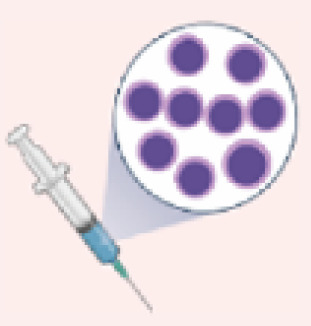	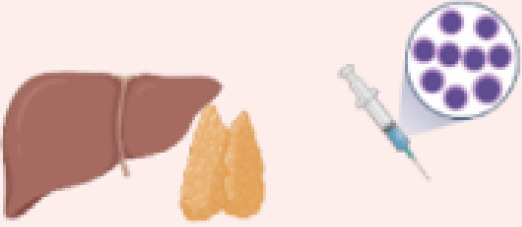	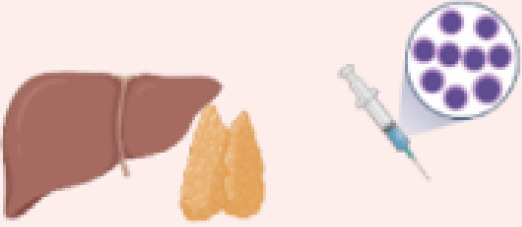
**Immune Reconstitution**	T cell engraftment Multilineage hematopoiesis No primary immune response	T cell engraftment No multilineage hematopoiesis No primary immune response	Multilineage hematopoiesis Primary immune response No HLA restriction	Multilineage hematopoiesis Primary immune response Human HLA T cell restriction	Multilineage hematopoiesis Primary immune response Human HLA T cell restriction
**References**	McCune Namikawa (11), Namikawa Weilbaecher (12)	Moiser, Gulizia (13), Hesselton. Greiner (14), van Rijn, Simonetti (15), Ali, Flutter (16)	Kamei-Reid and Dick (17), Peault, Weissman (18), Hiramatsu, Nishikomori (19), Danner, Chaudhari (20)	Lan, Tonomura (21), Melkus, Estes (22), Brainard, Seung (23), Stoddart, Maidji (24)	Lavender, Messer (25), Lavender, Pang (26), Lavander, Pace (27)

Created with BioRender.com.

The corrected **Table 1** appears below.

The authors apologize for this error and state that this does not change the scientific conclusions of the article in any way. The original article has been updated.

## Publisher’s Note

All claims expressed in this article are solely those of the authors and do not necessarily represent those of their affiliated organizations, or those of the publisher, the editors and the reviewers. Any product that may be evaluated in this article, or claim that may be made by its manufacturer, is not guaranteed or endorsed by the publisher.
